# The evolution of *intractable* Ménière’s disease: attacks resolve over time

**DOI:** 10.3389/fneur.2024.1469276

**Published:** 2024-10-24

**Authors:** F. R. Gerritsen, A. A. Schenck, H. Locher, R. van de Berg, P. P. van Benthem, H. M. Blom

**Affiliations:** ^1^Department of Otorhinolaryngology, Haga Hospital, The Hague, Netherlands; ^2^Department of Otorhinolaryngology and Head & Neck Surgery, Leiden University Medical Center, Leiden, Netherlands; ^3^The Novo Nordisk Foundation Center for Stem Cell Medicine (reNEW), Leiden University Medical Center, Leiden, Netherlands; ^4^Division of Vestibular Disorders, Department of Otorhinolaryngology and Head and Neck Surgery, School for Mental Health and Neuroscience (MHENS), Maastricht University Medical Centre, Maastricht, Netherlands; ^5^Department of Otorhinolaryngology, Antwerp University Hospital, Antwerp, Belgium

**Keywords:** Ménière’s disease, vertigo, vestibular system, endolymphatic sac surgery, evolution of disease

## Abstract

**Introduction:**

Knowledge of the natural and temporal course of a disease is important when deciding if an intervention is appropriate. In the case of Ménière’s disease (MD), there is some evidence that attacks diminish over time, but the topic remains controversial. A conservative approach to surgery is usually followed in northern Europe, and leads to strict patient selection before considering surgery. Here, we describe the evolution of vertigo attacks among a group of intractable MD patients in whom surgery was considered.

**Methods:**

Retrospective cohort study in a Ménière’s disease expert center. Patients with definite unilateral Ménière’s disease and persisting vertigo attacks despite treatment with intratympanic steroid injections were included. All patients had been waitlisted for participation in a planned trial assessing non-ablative surgery. They were waitlisted between June 2016 and June 2021 without undergoing the surgical intervention. In September 2022, data were collected from patient’s files and follow-up telephone interviews were conducted to assess the evolution of their vertigo attacks.

**Results:**

Thirty-five patients (54% male, mean age of onset 52 years, 51% right sided) were included in the analysis. Twenty-five patients (71%) eventually declined surgery. Of the 33 patients with complete information on vertigo attacks, 21 (64%) were free of vertigo attacks upon data collection, after a median disease duration of 5.3 years. Patients who did undergo surgery, had longer duration of disease than patients who did not.

**Discussion:**

Even in a population with intractable MD, most patients will experience relief of symptoms over time. On one hand, active treatment may accelerate relief of symptoms, but on the other hand, non-ablative therapies are of debatable effect and ablative intervention carries a risk of life long side effects. Therefore, any active intervention should be carefully considered.

## 1 Introduction

Ménière’s disease (MD) can be a highly incapacitating disease, characterized by spontaneous attacks of vertigo, sensorineural hearing loss and aural symptoms such as aural fullness and tinnitus (1–3). Treatment strategies aim to prevent, or at least reduce, the frequency and severity of attacks (4, 5). Usually, a stepwise approach is followed, starting with the least invasive and non-ablative therapies, such as diet and lifestyle intervention, medication such as diuretics or betahistine, and intratympanic (IT) injections with corticosteroids. However, effectiveness for these treatment modalities is uncertain (6–10). If symptoms persist despite the mentioned treatments, the disease can be considered “intractable” and several ablative treatment modalities can be considered. This includes intratympanic injection with gentamicin, selective neurotomy of the vestibular nerve and labyrinthectomy. These interventions seem successful in controlling vertigo attacks, but irreversibly destruct vestibular function and involve risk of permanent damage to the cochlear system (11, 12). A non-ablative, indisputable effective treatment with long-term effect is yet to be discovered. However, understanding of the disease’s evolution over time is essential in the process of developing new techniques.

Within the general MD population, the natural temporal evolution of vertigo attacks has been studied, albeit limited and with discordant outcomes. Moreover, it is important to realize that the patients in nearly every study were treated in some way, as it is considered unethical to completely refrain from treatment (13). This has led to a knowledge gap on the natural evolution of MD. Despite the lack of insight, several new treatment modalities were proposed in the past decades. In 2015, Saliba and colleagues introduced a novel surgical technique, referred to as “endolymphatic duct blockage” (EDB) (14). One prospective, controlled trial on this technique yielded high success rates of over 95% for EDB (14). However, the study was not blinded.

In Netherlands, treatments (both medical and surgical) require approval from the health care authorities before being allowed and reimbursed. In this decision, available literature is pivotal. Currently, EDB is a treatment that is not allowed as the publication of Saliba et al. contained methodological flaws. Therefore, a trial was designed to (re)assess effectiveness of EDB (15). As with every clinical trial, this study had a long startup phase, in which administrative, ethical and financial issues were addressed. In this period of approximately 5 years, patients were informed that the trial was upcoming. In all these patients, the disease was considered “intractable” because they did not experience relieve in symptoms despite treatment with medication (betahistine) and/or intratympanic injections with steroids. If patients were interested in participation in the trial, they were put on a waitlist. In the time between waitlisting and the start of the trial, the patients were regularly seen in the outpatient clinic and treated with regular treatments such as betahistine and intratympanic injections with steroids, depending on symptoms and patient’s preference. When the trial opened for inclusion, all patients were contacted and invited to (officially) participate in the trial. To the surprise of the researchers, the majority of patients declined participation.

The surprising refusal of most patients to undergo surgery led to further analysis of this group of patients, as presented here. This dataset provided an opportunity to study the evolution of unilateral MD in the (once) most severely affected patients, without surgical intervention. This could be of value for future counseling and research in MD patients, especially regarding this severely affected subgroup with intractable MD.

## 2 Methods

### 2.1 Study design

This study was a retrospective cohort study, conducted in an MD expert center. Data was extracted from patient files and complemented by a telephone interview. The protocol of this study was reviewed and approved by the Medical Research Ethics Committee Leiden-The Hague-Delft (number G21.186) and approved by the research board of the HagaHospital (number T21.102). Data collection took place in September 2022.

### 2.2 Patients and setting

Patients with MD were referred by their general practitioner or by otolaryngologists nationwide. If patients reported a high disease burden despite conservative treatment, they were informed about the upcoming EDB trial and information about the surgical interventions was provided. If patients met the inclusion criteria and provided verbal informed consent, they were waitlisted for participation in the trial. They were also informed that it was unknown when the trial would open for inclusion.

Inclusion criteria at the time of waitlisting were:

- Definite unilateral Ménière’s disease according to diagnostic criteria of the Bárány Society (1)
- More than three patient-reported attacks in the 6 months prior to waitlisting.
- Still suffering vertigo attacks despite non-surgical treatment (e.g., anti-vertigo medication, vestibular rehabilitation therapy, and dietary and lifestyle modifications), including at least two intratympanic (IT) injection each with corticosteroids (dexamethasone, methylprednisolone or triamcinolonacetonide).
- Age ≥ 18 years

Exclusion criteria:

- Severe disability (e.g., neurological, orthopaedic and cardiovascular), pregnancy or serious concurrent illness that might interfere with surgery or follow-up.
- Active additional neuro-otological disorders that may mimic MD [e.g., vestibular migraine, recurrent vestibulopathy, phobic postural vertigo, vertebrobasilar transient ischemic attacks (TIAs), vestibular schwannoma, congenital disorders, enlarged vestibular aqueduct (EVA) or genetic disorders (like DFNA9)].

**Figure 1:**
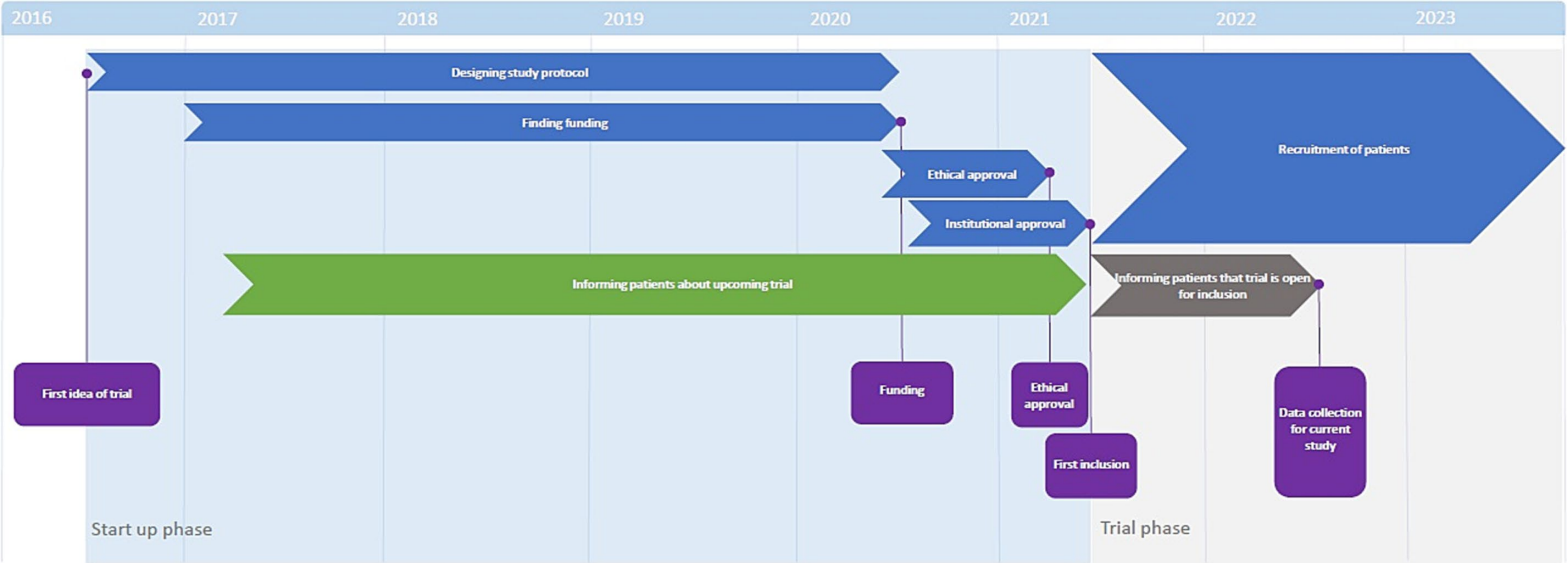
Timeline of trial preparation. The start-up phase lasted from 2016 to 2021. During this time, patients were informed that the trial was upcoming (green bar). When the study opened for inclusion mid-2021, patients were informed that the trial was open for inclusion (grey bar) and invited to participate.

The startup phase of the trial lasted from June 2016 until June 2021 (15). When recruitment for the trial started in June 2021, all patients on the waitlist were contacted and invited to participate in the trial. To assess evolution of attacks among these patients, they were contacted again in September 2022 for a telephone interview. A timeline of events can be found in Figure 1.

The primary outcome measure of this study was the proportion of patients that became free of vertigo attacks. “Free of vertigo attacks” was defined as the absence of vertigo attacks in the 6 months prior to data collection. The patients who were considered free of attack according to this definition, were collectively considered the attack-free population.

Secondary outcome measures were:

- The duration of disease, defined as the time between the first and last vertigo attack within the attack-free population.
- The proportion of patients that eventually underwent surgery.
- The therapeutic process prior to the (intended) surgery.
  - The median treatment period, defined as the time between the first visit at the otolaryngologist in our center and the moment of data-collection.
  - The median duration on the waiting list, defined as the time between the moment of waitlisting and the start of the EDB-trial.
  - The median number of consultations at the otolaryngologist.
  - The median amount of administered IT corticosteroid injections.
  - A description of other received therapies.
- The hearing evolution, represented by the hearing loss between first and last known pure tone audiogram, as endorsed by recent guidelines (16).
  - The mean bone conduction from the first and last known pure tone audiogram, calculated from the mean sensorineural pure-tone hearing threshold at 0.5, 1, 2 and 3 kHz.
  - The 3 kHz was calculated by averaging 2 and 4 kHz (17).
- The residual symptoms, defined as persisting audiovestibular symptoms in attack-free patients.

Lastly, a subgroup analysis was performed. Patients were categorized in groups of patients who had eventually undergone surgery, patients who had undergone ablative therapy and patients who had undergone neither of these treatment modalities. Outcomes were compared between the groups.

### 2.3 Statistical analysis

Statistical analyses were performed using SPSS version 28. For continuous and discrete variables, the mean and standard deviation were calculated in case of normal distribution, in other cases the median and interquartile range (IQR) were calculated. For categorical variables, frequencies and proportions were calculated.

For the subgroup analyses, the appropriate statistical test was chosen per outcome. All tests compared unpaired data. All numerical data were tested for normality. If numerical data had a normal distribution, an unpaired t-test was performed. If there was no numerical distribution among numerical outcome data, a Mann–Whitney U-test was performed. In case of binary or nominal categorical data, a Chi-square test was performed.

In case of missing data, no imputation was performed. In the outcomes, the number of data points was mentioned.

## 3 Results

### 3.1 Patient characteristics

From June 2016 until June 2021, 446 MD patients visited the MD expert center. Forty-three MD patients were waitlisted for participation in the EDB-trial. Upon the current analyses, eight of these patients were excluded because they developed bilateral MD (*n* = 1) or vestibular migraine (*n* = 1), had undergone less than two IT injections with corticosteroids (*n* = 5), or due to uncertainty concerning the diagnosis after further progress of the disease (*n* = 3). Two patients had overlap of exclusion criteria. Eventually, 35 patients met the inclusion criteria for this study and were included.

A small male predominance (54%) was observed and the mean age of onset was 52 years-old (SD 13 years). The side of disease was evenly distributed (18 right ears versus 17 left ears). The median duration of follow-up was 3.9 years (IQR 1.4 years).

### 3.2 Evolution of vertigo attacks

Thirty-three out of 35 patients agreed with the telephone interview, and data on vertigo attacks were complete for these patients. Two patients could not be reached. Sixty-four percent (21/33) of the included patients were free of attacks upon the interview. Additionally, two patients were not free of attacks but had sufficient control of symptoms with intratympanic steroid injections.

The median total duration of disease in the attack-free population was 5.7 years (IQR 4.2 years) from the first symptoms until the last attack.

### 3.3 Treatment and therapeutic process

**Figure 2:**
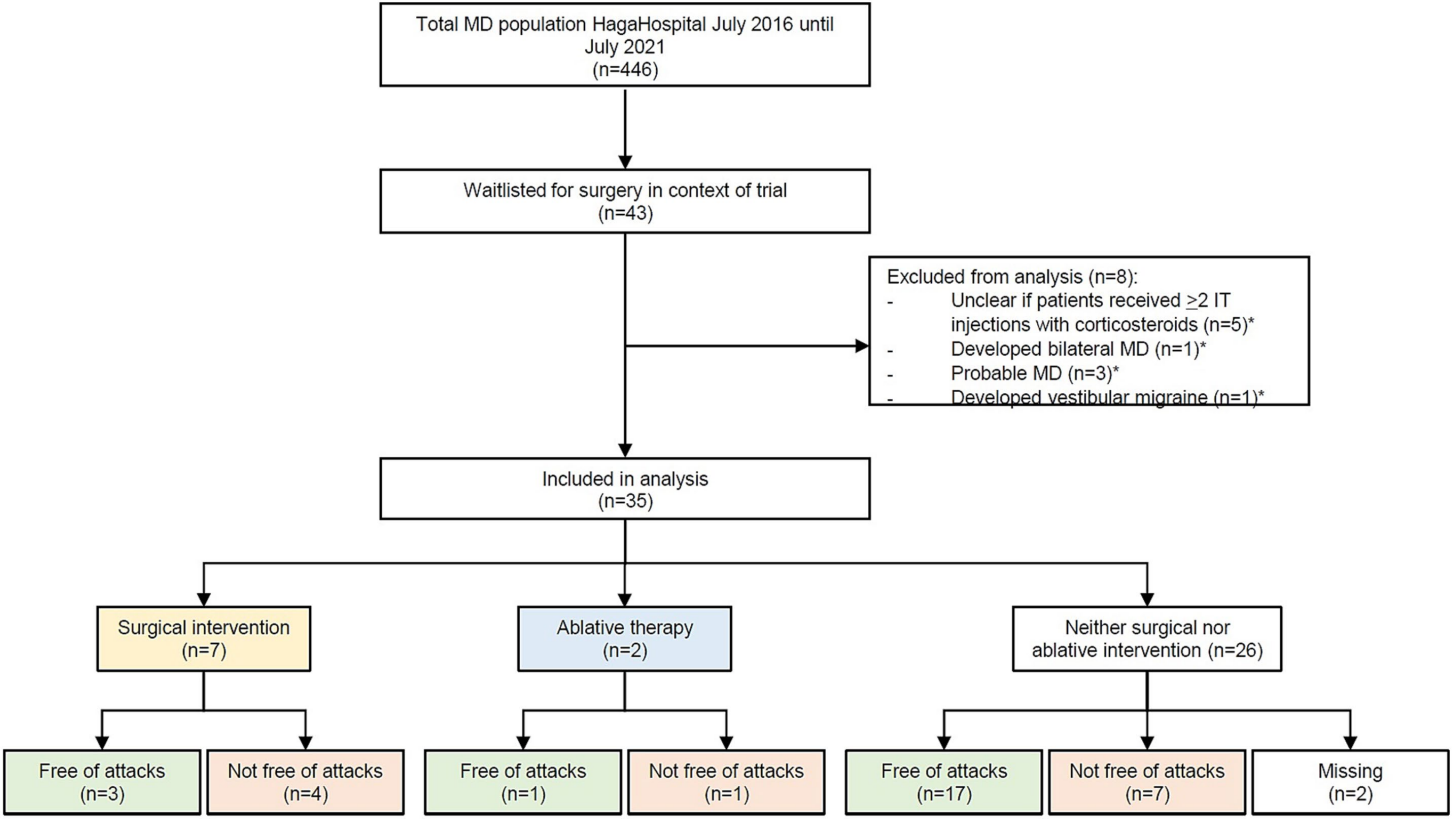
Flowchart of the patients that were included in this study. Four hundred and forty six patients were diagnosed with Ménière’s disease; 43 of these patients were put on the waiting list. Thirty-five patients were included in the eventual analysis. “Free of attacks” was defined as no attacks in the 6 months to data collection.

Of all 35 patients, data on surgical intervention were complete. Eighty percent (28/35) of the waitlisted patients did not undergo surgical intervention, after a median duration on the waiting list of 1.2 years (IQR 0.5). In 25 of 28 cases, this was the patient’s choice. Two patients were excluded from surgery by the surgeons due to language difficulties (*n* = 1) and previous radiotherapy at the surgery site (*n* = 1). One patient was not operated due to ambiguity in patient’s preferences. Seven patients eventually underwent surgical intervention, either in the context of the trial or abroad. A flow chart of the (sub)groups can be found in Figure 2.

Patients visited the otolaryngologist in the MD center for a median of 8 times (IQR 12) during a median treatment period of 2.4 years (IQR 3.4 years). The median amount of IT corticosteroids per patients was six (IQR 7 injections). Two patients underwent IT injections with gentamicin, 32 patients received anti-vertigo medication (betahistine, cinnarizine) and 18 patients were referred to a physiotherapist for vestibular rehabilitation therapy. All administered treatments can be found in Supplementary Table S1.

### 3.4 Hearing evolution and residual symptoms

From 28 patients, multiple audiograms were available. The mean duration between first and last audiogram was 32.8 months (SD 25.1 months). The mean bone conduction at the first PTA was −41.6 dB (*n* = 33, SD 22.7 dB), and −51.1 dB at the last PTA of the *affected* ear (*n* = 27, SD 15.7 dB). This was a significant reduction in hearing (*p* < 0.001). Of the *unaffected* ear, the mean bone conduction at the first audiogram was −13.3 dB (*n* = 33, SD 8.9), and − 15.7 dB at the last PTA (*n* = 27, SD 12.3). This difference was significant as well (*p* < 0.001), although the mean hearing loss was much smaller than in the affected ear (−10.5 dB versus −2.4 dB respectively).

The attack-free patients (*n* = 21) reported hearing loss (*n* = 20), tinnitus (*n* = 14), unsteadiness (*n* = 11) and aural fullness (*n* = 5) as residual symptoms.

### 3.5 Subgroup analysis: non-surgical treatment vs. surgical treatment

Patients were divided into the following subgroups:

- Patients who underwent surgical intervention (*n* = 7). All seven patients underwent non-ablative surgery, either in the context of the mentioned trial (EDB or endolymphatic sac decompressions), or abroad (EDB).
- Patients who underwent ablative therapy (*n* = 2). In both cases, this concerned intratympanic injection with gentamicin.
- Patients who underwent neither surgical nor ablative therapy (*n* = 26).

As the subgroup of ablative therapy comprised of only two patients, calculations were performed only on the other two groups. There was no difference in sex, age or side of disease among the groups. Moreover, the proportion of patients who were free of attacks was not statistically different between the groups (71% among the non-operated vs. 43% in the operated group, *p* = 0.46) (see Table 1a). Patients who underwent surgery, had longer duration of disease (2,465 days among the non-operated vs. 3,604 days among the operated patients, *p* = 0.04). This group also visited the ENT-consultant more often (15 vs. 8 times, *p* = 0.02) and had more telephonic consultations (11 vs. 4, *p* < 0.001) (Table 1a). The difference between the first and last PTA was not different between the groups (Table 1b). All outcomes and comparisons between the subgroups can be found in Table 1.

**Table 1:** Subgroup analysis, subdivided in characteristics (a) and hearing (b).

		Subgroup analysis				
	Total	No surgical/ablative therapy	Surgery	Ablative therapy	Appropriate statistical test	Subgroup comparison no invasive/ablative therapy *vs* surgery
	*n* = 35	*n* = 26	*n* = 7	*n* = 2		
*a. Characteristics*						
Sex					Chi square test (categorical binary outcome data)	*p* = 0.74
M	19 (54%)	13 (50%)	4 (57%)	2 (100%)		
F	16 (46%)	13 (50%)	3 (43%)	0 (0%)		
Side of disease					Chi square test (categorical binary outcome data)	*p* = 0.61
AD	18 (51%)	12 (46%)	4 (57%)	2 (100%)		
AS	17 (49%)	14 (54%)	3 (43%)	0 (0%)		
Outcome					Chi square test (categorical nominal outcome data)	*p* = 0.17
Free of attacks	21 (64%)	17 (71%)	3 (43%)	1 (50%)		
Not free of attacks	12 (36%)	7 (29%)	4 (57%)	1 (50%)		
Unknown	2	2	0	0		
Age at diagnosis (by ENT surgeon)					Unpaired t-test (numerical data with normal distribution)	*p* = 0.19
Mean (range)	51 (21–74)	49 (21–72)	52 (36–67)	73 (72–74)		
SD	13.3	13	10	1.4		
Duration of disease (days)	*n* = 33	*n* = 24			Mann–Whitney U test (numerical data with no normal distribution)	*p* = 0.04
Mean (range)	3,006 (243–12,889)	2,465 (243–8,507)	3,604 (1,903–6,633)	7,403 (1,916–12,889)		
SD	2,599	1919	1963	7,759		
Consults with ENT-consultant					Unpaired t-test (numerical data with normal distribution)	*p* = 0.02
Mean (range)	9 (1–29)	8 (1–20)	15 (5–29)	2 (1–3)		
SD	7	6	8	1		
Telephonic consults					Mann–Whitney U test (numerical data with no normal distribution)	*p* < 0.001
Mean (range)	6 (0–25)	4 (0–9)	11 (2–25)	4 (2–6)		
SD	5	3	7	3		
Number of IT injections					Mann–Whitney U test (numerical data with no normal distribution)	*p* = 0.78
Mean (range)	7 (2–20)	6 (2–17)	7 (2–18)	12 (3–20)		
SD	5	4	6	12		
*b. Hearing*						
Mean bone conduction first PTA (*affected* side) (dB)	*n* = 33	*n* = 25		*n* = 1	Unpaired t-test (numerical data with normal distribution)	*p* = 0.07
Mean (range)	−42 (−74 to −8)	−38 (−68 to −8)	−53 (−74 to −16)	−52		
SD	−19	−18	−19			
Mean bone conduction last PTA (*affected* side) (dB)	*n* = 27	*n* = 20	*n* = 6	*n* = 1	Unpaired t-test (numerical data with normal distribution)	*p* = 0.40
Mean (range)	−51 (−76 to −14)	−49 (−75 to −14)	−55 (−76 to −44)	−67		
SD	−16	−17	−11			
Difference (*affected* side) (dB)	*n* = 26	*n* = 20	*n* = 6	NA (different patients)	Unpaired t-test (numerical data with normal distribution)	*p* = 0.27
Mean (range)	−11 (−58 to +21)	−13 (−58 to +20)	−3 (−29 to +21)			
SD	−18	−18	−17			
Mean bone conduction first PTA (*unaffected* side) (dB)	*n* = 33	*n* = 25		*n* = 1	Mann–Whitney U test (numerical data with no normal distribution)	*p* = 0.56
Mean (range)	−13 (−36 to −2)	−13 (−316 to −2)	−15 (−36 to −3)	−14		
SD	−9	−9	−11			
Mean bone conduction last PTA (*unaffected* side) (dB)	*n* = 27	*n* = 20	*n* = 6	*n* = 1	Mann–Whitney U test (numerical data with no normal distribution)	*p* = 0.22
Mean (range)	−16 (−53 to −1)	−13 (−34.0 to −1)	−19 (−38 to −7)	−53		
SD	−12	−10	−11			
Difference (*unaffected* side) (dB)	*n* = 26	*n* = 20	*n* = 6	NA (different patients)	Unpaired t-test (numerical data with normal distribution)	*p* = 0.20
Mean (range)	−1 (−18 to +9)	0 (−18 to +9)	−4 (−11 to +5)			
SD	−6	−6.5	−6			
WRS first PTA (*affected* side)	*n* = 19	*n* = 16	*n* = 3		Unpaired t-test (numerical data with normal distribution)	*p* = 0.42
Mean (range)	77 dB (40–110)	75 dB (40–110)	85 dB (73–96)			
SD	18	19	12			
WRS last PTA (*affected* side)	*n* = 23	*n* = 16	*n* = 5		Unpaired t-test (numerical data with normal distribution)	*p* = 0.73
Mean (range)	82 dB (46–105)	81 dB (46–105)	84 dB (68–103)	85 dB (65–105)		
SD	17	18	16	28		
WRS difference (affected side)	*n* = 11	*n* = 10	*n* = 1			NA
Mean (range)	13 dB (−14 to 42)	13 dB (−14 to 42)	18 dB			
SD	19	20				
WRS first PTA (*unaffected* side)	*n* = 15	*n* = 12	*n* = 3		Unpaired t-test (numerical data with normal distribution)	*p* = 0.86
Mean (range)	46 dB (31–60)	46 dB (31–57)	45 dB (34–60)			
SD	9	8	13			
WRS last PTA (*unaffected* side)	*n* = 17	*n* = 12	*n* = 3		Unpaired t-test (numerical data with normal distribution)	*p* = 0.79
Mean (range)	45 dB (30–75)	43 dB (30–57)	45 dB (35–57)	57 dB (38–75)		
SD	12	10	11	26		
WRS difference (unaffected side)	*n* = 8	*n* = 6	*n* = 2		Unpaired t-test (numerical data with normal distribution)	*p* = 0.80
Mean (range)	1 dB (−10 to 17)	1 dB (−10 to 17)	−1 dB (−3 to 1)			
SD	9	10	3			

M, male; F, female; AD, right ear; AS, left ear; ENT, ear, nose, throat; SD, standard deviation; IT, intratympanic.

PTA, pure tone audiometry; dB, decibel; SD, standard deviation; WRS, word recognition score, the loudness at which 50% of the words was correctly heard by the patient.

## 4 Discussion

### 4.1 Evolution of disease

This study demonstrates that most patients (80%) who once opted for surgery as treatment for Ménière’s disease, refused surgery after a relatively short period of waiting (mean 1.2 years). Apparently, this period of waiting was enough for symptoms to diminish or even dissolve completely. During follow-up, 64% (*n* = 21/33) of the study population became free of vertigo attacks with a median duration of disease of 5.3 years (IQR 7.4 years).

As with every disease, knowledge of the natural course of the disease is imperative. Because of the intrusive nature of the disease, this is difficult to assess in MD: it could be considered unethical to refrain from any sort of treatment (13). This has led to the absence of data of patients who were not treated at all and in whom the “natural evolution” could be observed. The largest group of patients was reported by Perez-Garrigues, describing 510 patients who were treated only with oral medication (18). In the first years of disease, a rapid decline in number of vertigo attacks per year was seen, suggesting a benign evolution of MD. After 8 years, there was a stabilization in the decrease in attack frequency. Although it was stated that “most patients reach a vertigo-free state”, no exact percentages were reported. Perez-Carbonell and colleagues reported a similar pattern of decreasing attacks among 327 patients (19). Findings of Friberg et al. among 161 patients also supported this conclusion (20). Other studies suggested a less fortunate course with vertigo attacks even after long-term follow-up (21), although generally in reduced frequency (22). Thus, from the current body of literature, we can conclude that the general evolution of disease in patients suffering from MD is rather benign, with reduction of attacks over time.

However, in the current analysis, only patients with “intractable” disease were included; they suffered frequent attacks despite treatment with at least intratympanic corticosteroid injections (Supplementary Table S1). Comparison with a general MD population is therefore not appropriate, but there are some papers discussing the evolution of a subset of patients similar to the current population. Silverstein et al. report about a group of patients to whom surgery was offered, but was declined (23). After over 3 years of follow-up, 71% of the patients were free of attacks. This rate is comparable to the presented data. Green et al. report that 57% of this non-operated patients were free of vertigo attacks after 9 years of follow-up, a slightly smaller proportion than the present population (24). Sumi et al. report that 79% was free of vertigo after 10 or more years of follow-up, although three patients had undergone surgery (25).

Kerr and Toner reported a group of 23 patients whom were offered surgery (26, 27). Within 8 weeks, 12 patients experienced “dramatic improvement” and eventually refused surgery. They argue that this improvement might be due to the prospect of surgery, and that improvement after surgery, had they proceeded, should not be attributed to the surgery, but to *the idea* of surgery. Interestingly, the proportion of patients who improved in Kerr’s paper was very similar this study, a group that also faced the prospect of surgery.

Together, these papers suggest that outcomes for patients to whom surgery is offered, is just as good as for the general MD population.

### 4.2 Surgical intervention

From the total MD population of 446 patients in the current center, 35 patients were included in the final analysis and only seven patients eventually underwent surgery. This is 1.5% of our total population. In other studies, this percentage is usually higher, about 10–20% of the total MD population (19, 25, 28). Green et al. even reported surgery in 34% of his 119 patients (24). The fact that the proportion of operated patients was substantially lower in our population than in other papers, may be explained by the “northern Europe approach” of treatment. The attitude towards surgery is usually very conservative, and surgical intervention is generally considered a “last resort”. After the Danish RCT’s of Thomsen and Bretlau, the perspective on non-ablative surgery is even more reserved (29, 30). Due to the restrictive policy on surgery, there is a strict patient selection for surgery, as was also the case for our patients. Therefore, our population consisted only of the most severely affected patients. Among this subgroup of 35 patients, 7 (20%) eventually underwent surgery. This was similar to the numbers reported by Haid et al., who report that 15% of their patients with intractable disease underwent surgery (31). This German research group may have had the same conservative approach to surgery, leading to similar surgery rates. The fact that geographical location may affect the decision for surgery, makes it hard to compare the outcomes of this study with most of the available literature.

However, from these data, it can be concluded that even among the most severely affected patients (“proven” by the fact that they were selected for surgery), symptoms diminish over time, with a substantial proportion of patients reaching an attack-free state.

### 4.3 To treat or not to treat?

Regarding the outcomes of attacks, time seems to have a favorable effect on attacks, even in the most severely affected patients. Interestingly, similar to the studies on the natural evolution of the disease, nearly every study on *active* intervention for MD also reports this “success rate”. Supporting literature can be found for all sorts of interventions; conservative treatments such as lifestyle and dietary changes (32), diuretics (33), betahistine (34). For more invasive therapies, such as intratympanic injections with corticosteroids (35) and intravenous glycerol (36), similar supporting literature can be found. For surgical interventions, such as shunting of de endolymphatic sac (37), endolymphatic sac decompression (38, 39), endolymphatic duct blockage (14) or triple semicircular canal plugging (40), results are also similar with success percentages ranging from 60 to 96%.

In general, outcomes of treatment can be attributed to the actual effect of the investigated treatment, the natural evolution of the disease, the placebo effect, regression to the mean or to any combination of these factors. Yet, it is impossible to differentiate between the five. Therefore, depending on personal experience, attitude and beliefs, one could believe that “everything” helps, that “nothing” helps or, very selectively, that the treatment of interest helps. However, compared to placebo and in RCTs and systematic reviews, neither treatment has proven to be convincingly effective (7–10, 41). Moreover, the currently endorsed way of measuring treatment outcomes (42), comparing 6 months before intervention with the 18–24 months interval after intervention, carry the risk of measuring only the regression to the mean, potentially distorting outcomes in all studies that follow these guidelines.

The only exception may be ablative therapy, once described by a patient as “euthanasia of the sick ear”. Although there are no RCT’s comparing ablative surgical intervention such as labyrinthectomy or vestibular neurotomy to placebo surgery, there is some placebo-controlled evidence that intratympanic injection of gentamicin is an effective treatment, although limited (43, 44). The downside of any ablative therapy is the imminent damage that is inflicted on the vestibular system. In case of chemical ablation with gentamicin, it remains challenging to titrate the exact number of gentamicin injections necessary for effective treatment, and there may be an overshoot of iatrogenic vestibular hypofunction. In case of ablative surgery, vestibular areflexia is inevitable. Unilateral vestibular hypofunction leads to (at least) moderate handicap in over 30% of patients, even after vestibular rehabilitation therapy (45), meaning that patients might experience side effects for the rest of their lives. Another factor to take into account is the fact that MD may develop in the contralateral ear as well; in long term follow-up, bilateral disease is reported in up to 44% of the patients (20, 21, 46). With one labyrinth already afunctional as result of ablative therapy, patients may face complete deafness and serious balance and equilibrium problems. Therefore, ablative therapy should be very carefully considered.

In summary, non-ablative treatments are of debatable effect, ablative modalities carry a risk of life-long side effects, and, most importantly, there seems to be a decrease in attacks regardless of therapy. This leads to the question if patients with Ménière’s disease should ever be actively treated. Not doing so could protect patients from life-long treatment related side effects and save the health care system money by refraining from non-effective therapies. Lastly, it could save the patients false hopes and subsequent disappointment in case of relapse of attacks. On the other side, MD is known to have severe impact on quality of life (2). Any improvement may be very beneficial for the patient, even if “only” caused by the placebo effect. Although the placebo effect is thought to have a peak and carry-over effect (47, 48), this may be sufficient to suppress symptoms until the expected natural decline in symptoms.

The results of this study are limited by several factors. Firstly, the sample size is small (*n* = 35) and not all data are complete. This potentially affects generalizability of the results. The retrospective design of the study also poses a risk of (confounder) bias. Lastly, recall and non-response bias was potentially introduced by taking telephone interview. A prospective design could overcome these risks, and could also offer a broader look into relevant factor for patients with MD, such as quality of life. Ideally, such a study should be performed in a multicenter setting, to minimalize the risk of bias.

## 5 Conclusion

The current data support the statement that about 70% of the patients suffering from Ménière’s disease will experience a decline in symptoms, regardless of severity of disease and therapy. The current population was thought to suffer intractable disease, and yet most patients experienced relieve of symptoms in just over 1 year. Knowledge of the generally benign evolution of Ménière’s disease may be of value for patients and clinicians when weighing treatment options.

## Data Availability

The raw data supporting the conclusions of this article will be made available by the authors, without undue reservation.
