# Supplementary material for: The evolution of intractable Ménière’s disease: attacks resolve over time
Source: Front Neurol. 2024 Oct 24;15:1469276. doi: 10.3389/fneur.2024.1469276 (PMC11542254; doi:10.3389/fneur.2024.1469276)
Supplement: Supplementary file 1 [file Data_Sheet_1.PDF]

Additional Table 1. Overview of administered treatments of all patients.

Patient	Sex	Age*	ITS	ITG	Administered drugs	VRT	Other interventions	Subgroup	Free of attacks	Additional information
**1**	M	47	2	-	betahistin	Yes	Psychotherapy	Neither	Yes	
**2**	F	49	19	-	betahistin, cinnarizine, metoclopramide, domperidone	-	-	Surgery	No	Went abroad for surgery
**3**	M	42	3	-	betahistin	-	-	Neither	Yes	
**4**	M	32	4	-	cinnarizine	-	-	Neither	Yes	
**5**	M	21	4	-	betahistin, oxazepam, citalopram, cinnarizine, amitriptyline, ondansetran, domperidon	Yes	-	Neither	Yes	
**6**	F	69	8	-	betahistin, cinnarizine	-	-	Neither	No	Excluded for surgery: language difficulties
**7**	M	44	>10	-	cinnarizine	Yes	-	Neither	Yes	
**8**	F	60	3	-	oxazepam	-	-	Surgery	No	Participated in trial
**9**	F	36	2	-	betahistin, ondansetron	Yes	-	Surgery	Yes	Went abroad for surgery
**10**	F	46	2	-	betahistin, cinnarizine	Yes	-	Neither	No	Excluded for surgery: had undergone radiotherapy in surgery site in past
**11**	M	56	>5	-	betahistin, sertraline	Yes	-	Surgery	Yes	Participated in trial
**12**	M	48	13	-	betahistin, cinnarizine, metoclopramide, amitryptiline	-	-	Surgery	No	Went abroad for surgery
**13**	M	74	3	1	betahistin	Yes	Prism glasses	Ablative	Yes	
**14**	F	36	4	-	betahistin, domperidone, metoclopramide, amitriptyline	Yes	-	Neither	Yes	
**15**	M	67	>3	-	-	-	-	Neither	Yes	
**16**	F	50	10	-	betahistin, metoclopramide, domperidone, cinnarizine	Yes	-	Neither	No	
**17**	F	72	7	-	-	Yes	-	Neither	Yes	
**18**	F	53	17	-	betahistin	-	-	Neither	No	
**19**	M	72	20	2	betahistin	-	-	Ablative	No	Sufficient control of symptoms with ITC
**20**	M	67	6	-	cinnarizine, propranolol, betahistin	Yes	-	Surgery	Yes	Participated in trial
**21**	F	31	4	-	betahistin, metoclopramide, ondansetron	Yes	Psychotherapy	Neither	No	
**22**	M	67	6	-	betahistin	-	-	Neither	Yes	
**23**	M	34	7	-	meclizine, betahistin, cinnarizine, citalopram, cyclizine,	-	Prism glasses	Neither	Yes	

**24**	F	51	7	-	betahistin	Yes	-	Neither	Yes	
**25**	M	59	10	-	betahistin, cinnarizine, metoclopramide	-	-	Neither	No	Sufficient control of symptoms with ITC
**26**	M	60	4	-	betahistine, oral prednison (7 days)	Yes	Psychotherapy	Neither	No	
**27**	M	60	11	-	betahistin	-	-	Neither	Yes	
**28**	F	57	6	-	betahistin	-	-	Neither	Yes	
**29**	M	58	10	-	betahistin	-	-	Neither	Yes	
**30**	M	52	7	-	betahistin, cinnarizine	Yes	-	Surgery	No	Participated in trial
**31**	M	41	3	-	betahistin	-	-	Neither	Yes	
**32**	F	35	3	-	betahistin	Yes	-	Neither	Yes	
**33**	F	42	10	-	betahistin	Yes	-	Neither	Yes	
**34**	F	57	3	-	betahistin	-	-	Neither	Miss	
**35**	F	60	5	-	betahistin, cinnarizine	-	-	Neither	Miss	

* Age at diagnosis

ITS = intratympanic steroid injection, ITG = intratympanic gentamicin injection, VRT = vestibular rehabilitation therapy, - = no interventions
